# In-Network Processing of Skyline Join Queries in Wireless Sensor Networks Using Synopses of Skyline Attribute Value Ranges

**DOI:** 10.3390/s23063022

**Published:** 2023-03-10

**Authors:** Hyunchul Kang

**Affiliations:** School of Computer Science and Engineering, Chung-Ang University, Seoul 06974, Republic of Korea; hckang@cau.ac.kr

**Keywords:** skyline join query, wireless sensor network, sensor network database

## Abstract

We investigate the in-network processing of a skyline join query in wireless sensor networks (WSNs). While much research was conducted on processing skyline queries in WSNs, skyline join queries were dealt with only in traditional centralized or distributed database environments. However, such techniques cannot be applied to WSNs. Carrying out join filtering, as well as skyline filtering using them in WSNs, is infeasible due to limited memory in senor nodes and to excessive energy consumption in wireless communications. In this paper, we propose a protocol to process a skyline join query in WSNs energy efficiently with only a small amount of memory in each sensor node. It uses a synopsis of skyline attribute value ranges, which is a very compact data structure. The range synopsis is used both in the search of anchor points for skyline filtering and in 2-way semijoins for join filtering. We describe the structure of a range synopsis and present our protocol. To optimize our protocol, we solve some optimization problems. Through implementation and a set of detailed simulations, we show the effectiveness of our protocol. The range synopsis is confirmed to be compact enough for our protocol to work well with the limited memory and energy in each sensor node. For the correlated and random distributions, our protocol significantly outperforms other possible protocols, confirming the effectiveness of an in-network skyline as well as the join filtering capabilities of our protocol.

## 1. Introduction

**Figure 1:**
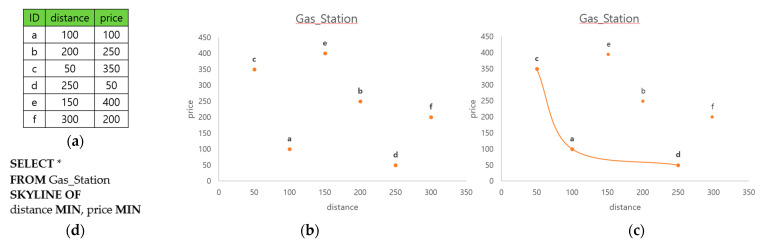
(**a**) Table Gas_Station; (**b**) attributes distance and price; (**c**) skyline points; and (**d**) skyline query in SQL.

In this paper, we investigate the in-network processing of a skyline join query in wireless sensor networks (WSNs). Skyline queries are widely used in multi-criteria decision making. The computation of the skyline was first studied in the maximum vector problem [1], and first addressed in the relational database context with the skyline queries [2]. For example, a car navigator is to find the gas stations that are close from a designated junction on a highway and with good price. Figure 1a shows an instance of the table Gas_Station that stores the distance and the price of the candidate stations. In Figure 1b, each station in Figure 1a is represented as a point in a 2-dimensional space, whose x and y dimensions are distance and price, respectively. Comparing two stations *a* and *b*, both the distance and the price of *a* are lower than those of *b*. In this case, *a* is said to dominate *b*. Comparing *c* and *d*, *c* is better than *d* in distance but worse than *d* in price. In this case, neither *c* nor *d* is said to dominate the other. The necessary and sufficient condition for a point *p* to dominate another point *q* in a 2-dimensional space is that (1) x and y values of *p* should not be worse than those of *q*, and (2) at least one of the x and y values of *p* should be better than that of *q*. The meaning of “better” depends on the application. A skyline point is the point not dominated by any other point, and the skyline is the line that connects the skyline points. Figure 1c shows that points *c*, *a*, and *d* are the skyline points. A skyline query is to find the skyline points. Using the SQL syntax proposed in [2], we can express the skyline query to find the gas stations in our example, as shown in Figure 1d.

There exist important variants of skyline queries, such as dynamic skyline query [3] and reverse skyline query [4]. The efficient processing of skyline queries and their variants attracted much attention. A comprehensive survey is given in [5]. A number of techniques were developed not just in centralized environments, but also in distributed and/or parallel environments [6], including the parallel solutions that take advantage of the MapReduce framework, such as Hadoop [7], and of the multicore architectures [8]. A heterogeneous architecture that employs both CPU and GPU for skyline computation on streaming data is investigated in [9]. A technique of efficient skyline recomputation after data updates in a big data environment is developed in [10]. The WSNs were also one of the major target environments of research, because the skyline query and its variants are very useful in monitoring applications [11,12,13,14,15,16,17,18,19,20,21,22,23].

**Figure 2:**
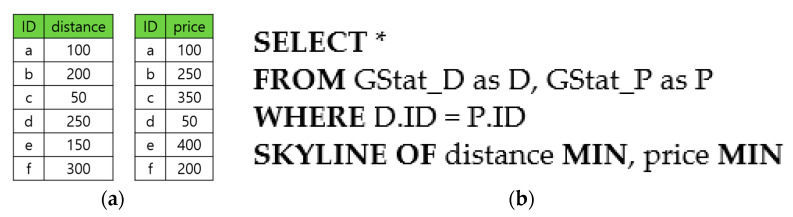
(**a**) Two tables GStat_D and GStat_P; and (**b**) skyline join query in SQL.

Another useful variant of skyline queries is the skyline *join* query [24,25,26,27,28,29,30,31,32,33,34,35]. In the example of Figure 1, the attributes distance and price are called skyline attributes. Given a skyline query, the relevant skyline attributes may not be stored in one table but distributed in multiple tables. Figure 2a shows that the distance and the price attributes in Figure 1 are separately stored in two tables: the distance in the GStat_D table and the price in GStat_P. A skyline join query is to find the skyline points when the skyline attributes are stored in multiple tables. In processing such a query, the join among the tables is necessary in addition to the dominance check among the tuples. We can express the skyline join query to find the gas stations in our example, as shown in Figure 2b.

Much work was conducted on the processing of skyline join queries in traditional centralized and distributed databases [24,25,26,27,28,29,30,31,32,33,34,35]. However, to our best knowledge, processing skyline join queries in WSNs was not addressed. Processing a skyline join query in WSNs does not seem to be feasible. In order to process a skyline join query, both skyline filtering, which eliminates those points dominated by some other point, and join filtering, which eliminates the tuples which do not have a join counterpart, need to be carried out. In traditional databases, efficient processing of these two tasks are possible, whereas in WSNs, it would not be the case. The reasons are as follows: First, the key element of skyline filtering in traditional databases is to sort the tuples of the join operand tables on the skyline attributes. In WSNs, however, in-network sorting of data is not feasible at all due to memory limitation in each sensor node. Second, the techniques developed for the join processing in WSNs mainly target the queries with low join rate [36]. This is because the higher the join rate is, the less effective in-network join filtering is. Considering that the join rate of a skyline join query could be 1.0 or very high, it would not be effective to employ the existing techniques for join processing in WSNs, such as those surveyed in [36].

In this paper, we suggest solutions to these fundamental problems, proposing a protocol for in-network processing of a skyline join query in WSNs. A core data structure for our protocol is devised, and related optimization problems for the optimal configuration of our protocol are solved. The effectiveness of the proposed protocol is shown through simulations. The contributions of this paper are as follows:

- We first establish an algorithm for processing skyline join queries in distributed databases, and then adapt it to WSNs, proposing a protocol. The algorithm for distributed databases conducts skyline filtering based on the sorting of tuples on skyline attributes. The adapted protocol constructs the synopses of skyline attribute value ranges instead of sorting the tuples. This range synopsis is a very compact data structure that could be stored in the limited memory of each sensor node. It consists of range descriptors and Bloom filters [37,38,39]. This structure is used not only for skyline filtering but also for join filtering based on 2-way semijoins [40,41,42];
- We address the optimization of the proposed protocol. The solutions for finding the optimal length of the Bloom filter in the range synopsis, and the optimal size of a range of the skyline attribute values is presented;
- We examine the threshold of the join rate, below which using join filtering only without skyline filtering is more efficient;
- Through implementation and a set of detailed simulations, we show the effectiveness of our proposed protocol with the random, correlated, and anti-correlated distributions [2];

The rest of this paper is organized as follows: In Section 2, the preliminaries for our work are presented. In Section 3, after an algorithm for processing a skyline join query in distributed databases is described, our protocol for WSNs and the range synopsis are described. In Section 4, the optimization of our protocol is addressed. In Section 5, the performance of our protocol is evaluated. In Section 6, related work is presented. Finally, in Section 7, the conclusions are drawn and the future work is given.

## 2. Preliminaries

In this section, the model of a WSN database and the query processing in WSNs is presented. The 2-way semijoin operator is described, and an overview of the Bloom filter with its theory is given.

### 2.1. Sensor Network Database and Query Processing

A wireless sensor network (WSN) is a network of sensor nodes called motes. They send and receive data among them through wireless communication. Each sensor node is a resource-constrained computing device and consists of an operating system, the memory, a processing unit, a wireless communication module, the power supply, and various sensors. The communication among the motes usually conforms to IEEE 802.15.4 standard. Although the range of wireless communication varies depending on the power of the mote, it is usually less than 100 m [43]. A WSN is usually connected to a server that is possibly connected to the Internet. It is called a *sink node*. A tree is typically used as a network topology [44]. The sink node could be the root of this routing tree. The protocol for processing a skyline join query we propose in this paper works in the WSNs using a tree topology.

**Figure 3:**
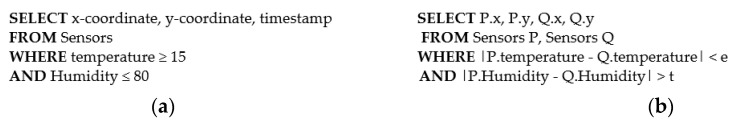
Examples of SQL-like queries in WSNs.

The values sampled by the sensors of each node can be stored in the memory of the node. Suppose each node is equipped with the sensors that measure temperature and humidity, for example. The stored values of temperature and humidity can be regarded as attribute values of a relational tuple, which could also include other attributes, such as node identifier, node location, measurement time, and so on. A set of tuples stored in all the sensor nodes can be modeled as a virtual table. That is, the WSN can be modeled as a relational database, and the data to be searched or monitored can be specified in a SQL-like declarative query language [44,45,46]. Figure 3a shows a query to retrieve the location and measurement time of the nodes with a temperature of 15 degrees or higher and a humidity of 80% or lower. The virtual table given in the FROM clause is named Sensors. Figure 3b shows a join query that searches the locations of two regions with similar temperature but a big difference in humidity.

A sensor network database can be viewed as a horizontally partitioned distributed database. It is an extremely partitioned one because the tuples of each table are distributed in each sensor node. The baseline method of processing a query in WSNs is for each node to transmit its tuple to the sink node and for the sink node to compute the query result. Data transmission is by multi-hop wireless communication along a path from each node to the root of the routing tree. Wireless communication is the dominant factor that consumes the limited energy in each sensor node. It is by orders of magnitude more costly than processing in a node [47]. For the longevity of WSNs, the query processing techniques should preserve energy. Thus, in-network filtering of data irrelevant to the query result is essential.

### 2.2. 2-Way Semijoin

The 2-way semijoin was proposed in [40] as an extension of the semijoin [48]. The semijoin is a core operation in processing join queries in distributed databases. For a join $R_{1}$ ${⋈}_{J=J}$ $R_{2}$, where $R_{1}$ and $R_{2}$ are stored at different sites of the network and *J* is the joining attribute, the semijoin $R_{1}{⋊}_{J=J}R_{2}$ is to fully reduce $R_{2}$ to $R_{2}^{\prime }$ in the sense that $R_{2}^{\prime }$ contains only the tuples of $R_{2}$ necessary for the join result. Sending $R_{2}^{\prime }$ instead of $R_{2}$ to the other site for the final join would save the communication cost. The semijoin $R_{1}{⋊}_{J=J}R_{2}$ is executed by sending $R_{1}$[*J*] (i.e., the projection of $R_{1}$ on *J*) to the site of $R_{2}$ and obtaining $R_{2}^{\prime }$ there with a natural join between $R_{1}$[*J*] and $R_{2}$.

A *2-way* semijoin $R_{1}⋊{⋉}_{J=J}R_{2}$ is to fully reduce $R_{1}$ as well as $R_{2}$. $R_{2}$ is reduced first by the semijoin $R_{1}{⋊}_{J=J}R_{2}$, and then $R_{1}$ is reduced subsequently. In the latter reduction, $R_{2}^{\prime }$[*J*] or $R_{1}$[*J*]$-R_{2}^{\prime }$[*J*], whichever is smaller in size, is sent back from the site of $R_{2}$ to that of $R_{1}$. This backward reduction is always cost-effective in communication.

### 2.3. Bloom Filter

The Bloom filter is a vector of *m* bits that can probabilistically represent a set of values *S* using *h* hash functions *H*_1_, ⋯, *H_h_* (*h* ≥ 1), each of which is to return an integer in the range [0, m−1] [37]. Initially, all the *m* bits are set to 0. For each value *v* in *S*, *H*_1_(*v*)-th, ⋯, *H_h_*(*v*)-th bits of the Bloom filter are set to 1. Given a value *w*, *w* is not in *S* if at least any one of the *H*_1_(*w*)-th, ⋯, *H_h_*(*w*)-th bits of the Bloom filter is 0. Otherwise, *w* is probably in *S*. When consulting a Bloom filter, false negative is not possible, but false positive is because of the collision in hashing. In the Bloom filter theory, the approximate probability *p* of a false positive is ${\left(1-e^{-(h\cdot k/m)}\right)}^{h}$, where *k* is the number of values in *S*, and *p* is minimized when h=(m/k)⋅ln2 [38,39].

## 3. Skyline Join Query Processing in WSNs

**Figure 4:**
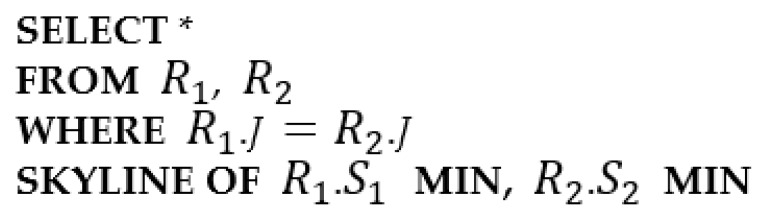
Query $Q_{j}$.

In this section, we propose a protocol for skyline join query processing between two tables, $R_{1}$ and $R_{2}$, that are stored in two regions of WSNs. The type of the skyline join query considered is query $Q_{j}$ in Figure 4. $R_{1}$ and $R_{2}$ have skyline attributes $S_{1}$ and $S_{2}$, respectively. They have a join key *J* as a common attribute. It is assumed that the values in $R_{1}$.*J* are unique, and the same for $R_{2}$.*J*. The join rate of $R_{1}$ and of $R_{2}$ is less than or equal to 1.0. The proposed protocol uses the properties described in Observation 1.

**Figure 5:**
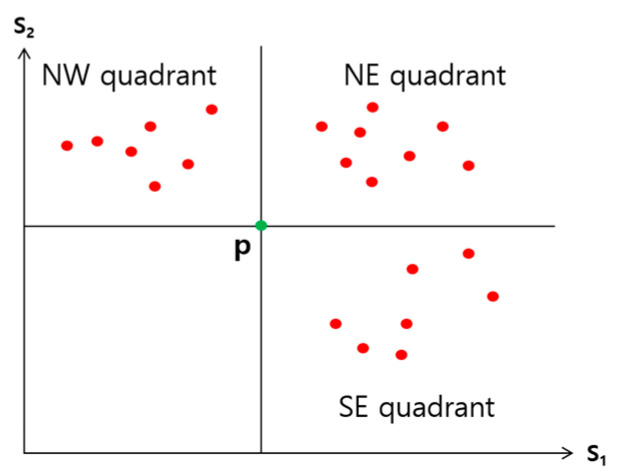
Anchor point.

Observation 1. Consider a point *p* in a 2-dimensional space whose *x* and *y* dimensions represent the skyline attributes $S_{1}$ and $S_{2}$, respectively (see Figure 5). This 2-dimensional space can be partitioned into four quadrants by the horizontal and vertical lines that run through *p*. (1) Since all the points contained in the NE quadrant are dominated by *p*, the points in the NE quadrant cannot be skyline points. (2) Any point in the NW quadrant does not dominate any in the SE quadrant, and vice versa. 

The point *p* in Observation 1 is called an anchor point [31,32,33]. Given $Q_{j}$ in vertically partitioned distributed databases, there is an algorithm that can conduct skyline filtering after finding an anchor point [32]. The data distribution assumed for the algorithm of [32] is based on vertical partitioning of a table that includes skyline attributes and the tuple identifier. It is assumed that the table is fragmented vertically, and each fragment is stored in a different site of a distributed database. Each fragment stores one skyline attribute of the table along with the tuple identifier as the join key. The tuple identifier is included in every fragment to enable the reconstruction of the original table by joining all the fragments. The join rate is 1.0.

In Section 3.1, we present an algorithm SKYJ_D_ that processes $Q_{j}$ in a distributed database based on the algorithm of [32]. In Section 3.2, we propose a protocol SKYJ_W_ that is devised by adapting SKYJ_D_ to WSNs.

### 3.1. SKYJ_D_: Skyline Join Query Processing in Distributed Databases

SKYJ_D_ is designed based on the algorithm of [32]. However, SKYJ_D_ does not assume that$\;R_{1}$ and $R_{2}$ of $Q_{j}$ are created by the vertical partitioning assumed in [32]. While the algorithm of [32] conducts no join filtering, SKYJ_D_ conducts join filtering in addition to skyline filtering to deal with the case where the join rate is less than 1.0. For this, 2-way semijoins [40,41] are employed.

In a distributed database where $R_{i}$ is stored at site *i*, *i* = 1, 2, suppose the result of query $Q_{j}$ is requested at site 3. The skeleton of SKYJ_D_ is as follows:

1. Site 3 requests site *i* to sort the tuples of $R_{i}$ on $S_{i}$ in increasing order, *i* = 1, 2;
2. Site 3 requests site *i* to send the join key of the first tuple, *i* = 1, 2;
3. Site 3 checks if a join match exists between the join keys of $R_{1}$ and $R_{2}$ received so far. Suppose that the join key received last is *v*, and it is received from site *i*, *i* = 1 or 2. If one of the join keys received so far from the other site (i.e., site *j*, where *j* = 3−i) is equal to *v*, a join match exists. If no join match is found, site 3 requests site *j* to send the join key of the next tuple. If site *j* has no tuple left, site 3 requests site *i* to send the join key of the next tuple. This step is repeated until a join match is found;
4. If a join match is found for join key *v*, let $JF_{i}$ be the set of all the join keys received from site *i* earlier than *v*, *i* = 1, 2. Site 3 obtains $JF_{1}^{\prime }$ and $JF_{2}^{\prime }$ using 2-way semijoins $JF_{1}⋊{⋉}_{J=J}R_{2}$ and $JF_{2}⋊{⋉}_{J=J}R_{1}$ so that $JF_{1}^{\prime }$ = $R_{2}$ ${⋊}_{J=J}$ $JF_{1}$ and $JF_{2}^{\prime }$ = $R_{1}$ ${⋊}_{J=J}$ $JF_{2}$, making the join filter *JF*, which is obtained as {*v*} ∪ $JF_{1}^{\prime }$ ∪ $JF_{2}^{\prime }$;
5. Site 3 sends *JF* to site *i*, requesting all the tuples of $R_{i}$ whose join keys are in *JF*, *i* = 1, 2;
6. Site 3 computes the final query result with the tuples received from site *i*, *i* = 1, 2.

If a join match exists for join key *v* in step 3, the pair ($w_{1}$, $w_{2}$) of $S_{1}$ and $S_{2}$ attribute values of the two tuples of $R_{1}$ and $R_{2}$, whose join keys are *v,* is the coordinate of the anchor point. By Observation 1, with only those tuples of $R_{1}$ and $R_{2}$, whose join keys are *v* or those sent from sites 1 and 2 earlier than *v*, the query result can be obtained. Out of those tuples, the ones without a join counterpart need to be filtered. This is conducted using 2-way semijoins in step 4. In step 5, only those tuples to be joined are requested, and in step 6, the final query result is obtained as follows: A join is performed between the received tuples of $R_{1}$ and $R_{2}$, and the dominance relationship is checked among the joined tuples, and only those not dominated by any other are included in the query result. In the checking of dominance relationship, the joined tuples in the NW quadrant of Figure 5 need to be checked only among themselves. The same for those in the SE quadrant.

### 3.2. SKYJ_W_: Proposed Protocol for Skyline Join Query Processing in WSNs

In this section, we present our protocol SKYJ_W_. First, the problems when applying SKYJ_D_ directly to WSNs, and the requirements that SKYJ_W_ must meet, are explained. The range synopsis, the core data structure of SKYJ_W_, and the execution process of SKYJ_W_ are described next in detail. The optimization of SKYJ_W_ is addressed in Section 4.

#### 3.2.1. Problems and Requirements

It is not feasible to apply SKYJ_D_ in WSNs. In a distributed database, $R_{1}$ and $R_{2}$ are stored in different sites as a table, while in WSNs, $R_{1}$ and $R_{2}$ are virtual tables, and the tuples belonging to each table are distributed in different nodes. Some of the problems are as follows: First, the sorting of the tuples of $R_{1}$ and $R_{2}$ on skyline attributes in step 1 of SKYJ_D_ corresponds to the sorting of the tuples of the virtual tables $R_{1}$ and $R_{2}$ in WSNs. This sorting is not realistic in WSNs due to the memory constraint of each sensor node. Second, the 2-way semijoins to filter the tuples with no join counterpart in step 4 would incur formidable communication overheads if conducted in WSNs. Furthermore, the effect of join filtering will be little if the join rate is high. Third, the search and transmission of tuples in step 5 would be very costly if executed in WSNs. When those tuples are searched, the sensor node where each of those tuples is stored needs to be identified. The communication overheads for that would be very high.

The requirements in processing skyline join queries in WSNs are as follows: First, it should be possible to efficiently find the anchor point despite memory constraints of sensor nodes. It should not resort to the conventional sorting. Second, it should be possible to efficiently conduct in-network join filtering with an acceptable amount of communication, even if the join rate is very high. Third, it should be possible to search for the tuples to be sent from each region of WSNs to the sink node with little communication overhead.

#### 3.2.2. Range Synopsis

In order to meet the requirements described at the end of Section 3.2.1, we use summary information on the skyline attribute value range. Let us call it a *range synopsis*. If we divide the domain of the skyline attribute $S_{i}$ of $R_{i}$ into *k* intervals of the same size, then each interval corresponds to a range of values of $S_{i}$, *i* = 1, 2. Each range is given an identifier from 1 to *k*. Given a skyline attribute value *w*, the function *rID*(*w*) returns the ID of the range to which *w* belongs. As in the experiments in [2], if *w* is in [0, 1), *rID*(*w*) is 1+⌊w⋅k⌋.

**Figure 6:**
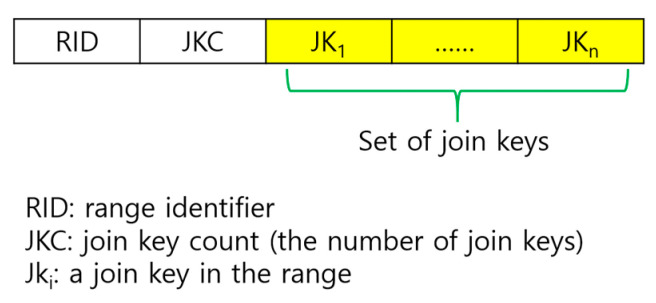
Range descriptor.

**Figure 7:**
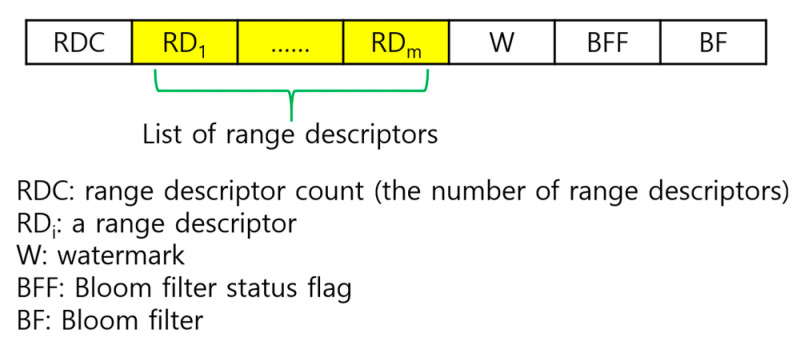
Range synopsis.

Each tuple sampled in each node in a region of WSNs belongs to a certain range according to its skyline attribute value. Given a set of tuples that belong to the same range, we summarize their information in a structure called *range descriptor*. Its structure is shown in Figure 6. It consists of the range ID, the number of join keys in the tuples, and the set of those join keys. A range synopsis is a collection of range descriptors constructed under the memory constraint of sensor nodes as follows: Given a set of tuples, the tuples are grouped by their skyline attribute value range, and all the range descriptors generated out of these groups are collected and stored in ascending order of the range ID. If memory overflow occurs (i.e., the memory space allocated per node for storage of these range descriptors is exceeded), some range descriptors are deleted and only its join keys are inserted into the Bloom filter (BF). The data structure of a range synopsis is shown in Figure 7. It consists of the number of range descriptors, the list of range descriptors, the watermark, the BF flag, and the BF. The BF flag indicates the usage status of the BF. The watermark is the upper bound of the range IDs that can appear in the list of range descriptors. That is, a range descriptor with its range ID exceeding the watermark cannot be stored in the list of range descriptors. Rather, only its join keys are inserted into the BF.

Given a query $Q_{j}$, a routing tree is constructed in the regions of $R_{1}$ and $R_{2}$, respectively. Let $r_{i}$ denote the root node of the routing tree in the region of $R_{i}$, *i* = 1, 2. Each node *n* in the routing tree stores a range synopsis for the set of tuples stored in all the nodes of the subtree whose root is *n*. Let us call it a *subtree synopsis*. In particular, we call the subtree synopsis of $r_{i}$ a *region synopsis*, *i* = 1, 2.

#### 3.2.3. Overview of SKYJ_W_

Given a query $Q_{j}$, $r_{1}$ and $r_{2}$ cooperate with a node $\tilde{m}$ located in the middle of the two regions in WSNs to conduct skyline filtering and join filtering. Suppose the query result is requested at the Section 3.2.9.

1. Initially, $\tilde{m}$ requests $r_{i}$ to construct the region synopsis of $R_{i}$, *i* = 1, 2;
2. $\tilde{m}$ requests $r_{i}$ to send the first range descriptor in the region synopsis of $R_{i}$, *i* = 1, 2;
3. $\tilde{m}$ checks if one or more join matches exist between the join keys of $R_{1}$ and $R_{2}$ contained in the range descriptors received so far (in a range pair between a range descriptor *d* of $r_{1}$ and *e* of $r_{2}$, if one of the join keys in *d* is equal to one in *e*, a join match exists). If no join match is found, $\tilde{m}$ requests $r_{i}$ to send the next range descriptor, *i* = 1, 2. This step is repeated until at least one join match is found or all the range descriptors in both of the region synopses of $R_{i}$ are exhausted, *i* = 1, 2;
4. If no join match is found, $\tilde{m}$ requests $r_{i}$ to update the region synopsis of $R_{i}$ and send the first new range descriptor in the updated region synopsis, *i* = 1, 2. After that, step 3 is resumed;
5. If one or more join matches are found, $\tilde{m}$ conducts skyline filtering using the anchor points associated with the join matches. Additionally, $\tilde{m}$ conducts join filtering with $r_{i}$ using 2-way semijoins that access the region synopsis of $R_{i}$ instead of $R_{i}$, *i* = 1, 2. $\tilde{m}$ makes the join filter *JF*, which consists of the join keys that survived, and sends *JF* to $r_{i}$, requesting the tuples of $R_{i}$ whose join keys are in *JF* to be sent to the sink node, *i* = 1, 2;
6. $r_{i}$ searches for the requested tuples by consulting the subtree synopses in the region of $R_{i}$ in the level order down the routing tree from $r_{i}$ to its descendants, and sends them to the sink node, *i* = 1, 2;
7. The sink node computes the final query result with the tuples received.

#### 3.2.4. Initial Construction of a Region Synopsis

Each node *n* of the routing tree in each region receives from its parent a command of constructing a range synopsis, broadcasting the command to its children unless *n* is a leaf node. For a tuple *t* sampled by *n*, *n* initializes the range descriptor for *t* as <*rID*(*w*), 1, {*v*}> provided that the skyline attribute = *w* and the join key = *v*. Now, each leaf node initializes its subtree synopsis as follows: the number of range descriptors = 1, the list of range descriptors = {<*rID*(*w*), 1, {*v*}>}, the watermark = the total number of value ranges for the skyline attribute, and the BF flag = *NU* (not used). It is sent to the parent. The parent node *p* merges the children’s subtree synopses and reflects the range descriptor for its own tuple, producing its own subtree synopsis. When merging two range descriptors <$id_{1}$, *a*, $J_{1}$> and <$id_{2}$, *b*, $J_{2}$>, if their range IDs are the same (i.e., $id_{1}$ = $id_{2}$), a range descriptor <$id_{1}$, a+b, $J_{1}\cup J_{2}$> is produced. Otherwise (e.g., $id_{1}<id_{2}$), a list of range descriptors {<$id_{1}$, a, $J_{1}$>, <$id_{2}$, b, $J_{2}$>} sorted in ascending order of the range ID is produced. In case of memory overflow in *p*, the BF is initialized and the BF flag is set to *U* (used). Among the range descriptors, the one with the largest range ID is deleted after its join keys are inserted into the BF. This means that the BF is to summarize the join keys of the range descriptors that cannot be contained in the list of range descriptors. Such deletion is continued until the memory overflow is resolved, after which the watermark is updated to c−1, where *c* is the range ID of the range descriptor deleted last. The watermark is to prevent the generation of an *incomplete* range descriptor. A range descriptor is incomplete if some of the join keys of all the tuples belonging to the range are missing in its set of join keys in the region synopsis. For example, suppose a certain range descriptor is deleted while the subtree synopsis of a node *n* is constructed. If the corresponding range reappears for the tuple *t* sampled by an ancestor node of *n* in the routing tree, and a new range descriptor *d* is created for *t*, *d* would be incomplete because the join keys inserted into the BF at *n* are missing in *d*. Therefore, a range descriptor with its ID exceeding the watermark cannot be included in the list of range descriptors. Rather, its join keys should be inserted into the BF. If a node *p* receives at least one subtree synopsis with the BF flag = *U* from its children, *p* sets its BF flag to *U*, initializing its BF to be the result of the ORing the children’s BFs, with the BF flag = *U*. Each node sends its subtree synopsis to its parent, and this process is continued until the subtree synopsis at the root, which is the region synopsis, is constructed.

#### 3.2.5. Additional Collection of Range Descriptors (Update of a Region Synopsis)

If no anchor point is found after checking all the pairs of range descriptors collected in the two region synopses, $\tilde{m}$ instructs $r_{i}$ to update its region synopsis by collecting additional range descriptors, *i* = 1, 2. In this update, only those range descriptors whose range ID is greater than the maximum range ID in the previous region synopsis are collected.

At the request of $\tilde{m}$, $r_{i}$ broadcasts a command for additional collection of such range descriptors to its children. Receiving this command, a node *n* checks its BF flag. If BF flag = *U*, *n* broadcasts the command to its children. Otherwise, *n* selects the qualified range descriptors in its own subtree synopsis, makes a subtree synopsis only with the selected ones, and sends it to its parent *p*. To update its subtree synopsis, *p* reflects the subtree synopses of its children and also reflects the range descriptor for its own tuple if it is qualified. In case of memory overflow, the BF is initialized and the BF flag is set to *U*. Among the range descriptors already collected for the previous region synopsis, the one with the smallest range ID is deleted after its join keys are inserted into the BF. If the memory overflow is not yet resolved after all such deletions, the same overflow handling process as in the initial construction of the region synopsis is applied to the additionally collected range descriptors. This process is continued until the region synopsis at the root is updated. The process of finding the anchor points with the new range descriptors is the same as that with the initial region synopses. The update of region synopses may be repeated multiple times until one or more anchor points are found.

#### 3.2.6. Skyline Filtering Using Anchor Points

**Figure 8:**
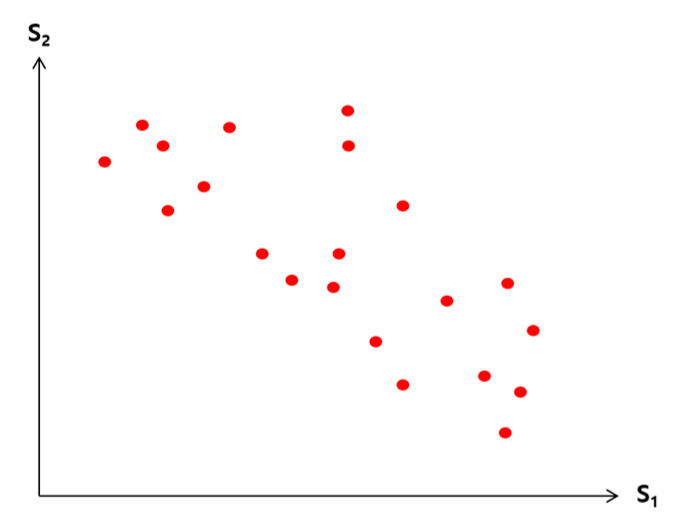
($S_{1},S_{2}$) value pairs. Each point corresponds to a join match.

In Figure 8, the x and y dimensions are for the values of the skyline attributes $S_{1}$ of $R_{1}$ and $S_{2}$ of $R_{2}$, respectively. Each point in this space, a pair of ($S_{1}$, $S_{2}$) values, represents a join match between a tuple of $R_{1}$ and that of $R_{2}$.

**Figure 9:**
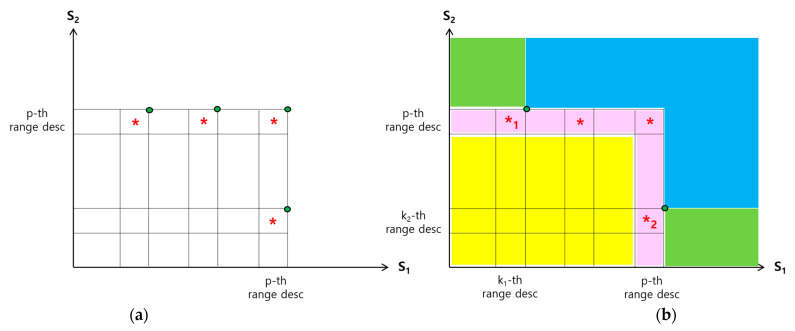
Skyline filtering using anchor points. (**a**) Join matches in one or more range pairs. In each of the range pairs marked with *, one or more join matches are found. The green dots are possible anchor points. (**b**) Skyline filtering using two anchor points. The green dots are the anchor points employed.

Figure 9a is to show the following, which happened during the search for the anchor points: (1) A total of *p* range descriptors were sent from $r_{1}$ to $\tilde{m}$, and also *p* range descriptors from $r_{2}$ to $\tilde{m}$. (2) There was no join match found in a total of (p−1)( p−1) range pairs with the first, second, …, (p−1)-th range descriptors from $r_{1}$, and those p−1 range descriptors from $r_{2}$. (3) At least one join match is found in each of the range pairs marked with * out of all the examined range pairs between the *p*-th range descriptor from $r_{1}$ and each of the *p* range descriptors from $r_{2}$. (4) At least one join match is found in each of the range pairs marked with * out of all the examined range pairs between the *p*-th range descriptor from $r_{2}$ and each of the *p* range descriptors from $r_{1}$.

Unless a join match does not exist, there is at least one range pair with one or more join matches out of the $p^{2}$ range pairs, and the number could be more than one. In the example of Figure 9a, there are four such range pairs. There could be multiple join matches in each of the range pairs marked with *.

With a join match for a join key *v*, the point at the coordinate ($w_{1}$, $w_{2}$), where $w_{i}$ is the value of $S_{i}$ in the tuple of $R_{i}$ with *J* = *v*, can be used as an anchor point, *i* = 1, 2. However, since *v* is given through a range descriptor, $w_{i}$ is not exactly known. Only the range to which $w_{i}$ belongs is known. Thus, a possible anchor point for conducting skyline filtering, while ensuring the accuracy of the query result is the one at the coordinate which corresponds to the upper right corner of the rectangle representing the range pair marked with * in Figure 9a. There are four green dots in Figure 9a. Each of them denotes such an anchor point.

Figure 9b shows the four areas around the two selected anchor points denoted in Figure 9a: (1) Points in the blue area do not need to be considered in query processing by skyline filtering. (2) There are no points in the yellow area. (3) In the pink area, the points exist only in those range pairs marked with *. They correspond to the join matches and should be considered in query processing. (4) There may be points in the green area and should be considered in query processing.

In Figure 9b, the anchor points employed for skyline filtering are the two points for the range pairs marked with *_1_ and *_2_. One is with the minimum $S_{1}$ value, and another with the minimum $S_{2}$ value among all the range pairs marked with *. For the anchor point employed with a minimum $S_{i}$ value, let the corresponding range pair be between the $k_{i}$-th range descriptor sent by $r_{i}$ and the last range descriptor sent by the other root node $r_{j}$, where *i* = 1, 2 and *j* = 3−i. Then, all the join keys for the join matches found are relevant to query processing. Let *A* be the set of such join keys. Let $JF_{i}$ be the set of all the join keys contained in the first $k_{i}$ range descriptors received from $r_{i}$ except those in *A*, *i* = 1, 2. Those in $JF_{i}$ are also relevant, *i* = 1, 2. Let *JF* be *A* ∪ $JF_{1}$ ∪ $JF_{2}$. The query result can be computed only with the tuples whose join keys are in *JF*. If the join rate is 1.0, $\tilde{m}$ sends *JF* to $r_{1}$ and $r_{2}$ to search for such tuples in the regions of $R_{1}$ and $R_{2}$.

#### 3.2.7. Join Filtering Using 2-Way Semijoins

When the join rate is less than 1.0, some tuples with join keys in *JF* do not have a join counterpart, thus, efficient in-network join filtering is required. Let $JF_{i}^{\prime }$ be the set of all the joinable keys in $JF_{i}$, *i* = 1, 2. Since the join keys in $JF_{i}$ were received from the region of $R_{i}$, $\tilde{m}$ can obtain $JF_{i}^{\prime }$ using 2-way semijoins$\;JF_{1}⋊{⋉}_{J=J}R_{2}$ and $JF_{2}⋊{⋉}_{J=J}R_{1}$ by sending $JF_{i}$ to the other region of $R_{j}$, *i* = 1, 2 and *j* = 3−i. That is, $JF_{1}^{\prime }$ = $R_{2}$ ${⋊}_{J=J}$ $JF_{1}$ and $JF_{2}^{\prime }$ = $R_{1}$ ${⋊}_{J=J}$ $JF_{2}$, *i* = 1, 2.

Observation 2. For a node *n* of the routing tree, let $T_{n}$ be the subtree whose root is *n*. In order to probabilistically determine whether a tuple $t_{u}$ having a join key *u* exists in $T_{n}$, only *n* needs to be accessed. In the subtree synopsis of *n*, if *u* exists in the set of join keys in some range descriptor, $t_{u}$ exists in $T_{n}$. If *u* does not exist in the set but the result of consulting the BF with *u* is positive, $t_{u}$ may exist in $T_{n}$. In this case, if the probability of a false positive for the BF is very low, the probability that $t_{u}$ exists in $T_{n}$ is very high. 

According to Observation 2, the above 2-way semijoins can be executed by accessing the region synopsis of $R_{i}$ instead of $R_{i}$, *i* = 1, 2. That would significantly save the communication cost. The process of obtaining $JF_{1}^{\prime }$ in the region of $R_{2}$ is as follows: $r_{2}$ receives $JF_{1}$ from $\tilde{m}$. For each join key *u* in $JF_{1}$, if *u* is in any set of the join keys in the region synopsis of $R_{2}$, *u* is included in $JF_{1}^{\prime }$. Otherwise, if the result of consulting the BF with *u* is positive, *u* is included in $JF_{1}^{\prime }$.

We see that $JF_{1}^{\prime }$ may include non-joinable keys due to false positives of the BF. However, it does not undermine the accuracy of the query result. In the subsequent process of searching for the tuples of $R_{2}$ whose join keys are in $JF_{1}^{\prime }$, the join key due to a false positive is finally filtered because it will turn out that no corresponding tuple exists. 

Additionally, $\tilde{m}$ obtains $JF_{2}^{\prime }$ symmetrically, and $\tilde{m}$ sends *A* ∪ $JF_{1}^{\prime }$ to $r_{1}$. Since $r_{1}$ already has $JF_{2}^{\prime }$ as the result of the previous 2-way semijoin, $r_{1}$ now obtains $JF^{\prime }=$ *A* ∪ $JF_{1}^{\prime }$ ∪ $JF_{2}^{\prime }$, and $r_{2}$ also obtains $JF^{\prime }$ symmetrically. The query result can be computed only with the tuples whose join keys are in $JF^{\prime }$, and $r_{i}$ is ready to search for such tuples in the regions of $R_{i}$, *i* = 1, 2.

#### 3.2.8. Tuple Search and Transmission

In the regions of $R_{1}$ and $R_{2}$, those tuples whose join keys are in $JF^{\prime }$ are searched and sent to the sink node. This process is efficiently conducted by Observation 3:

Observation 3. For a node *n* of the routing tree, let $T_{n}$ be the subtree whose root is *n*. Suppose that a tuple $t_{u}$ with a join key *u* does not exist in $T_{n}$. It can be confirmed by accessing only *n*, without accessing all the nodes of $T_{n}$. In the subtree synopsis of *n*, if (1) there is no *u* in any set of the join keys in the range descriptors, and (2) either the BF flag = *NU* or the result of consulting the BF with *u* is negative, $t_{u}$ does not exist in $T_{n}$. 

Each node *n* in the regions of $R_{1}$ and $R_{2}$ executes the following four steps: (1) *n* receives a set of join keys *L* from its parent (if *n* is the root, $JF^{\prime }$ is given as *L*). (2) If the join key *u* of the tuple *t* sampled in *n* is in *L*, *n* sends *t* to the sink node and deletes *u* from *L*. (3) For each join key *v* in *L*, if it is confirmed by Observation 3 that no tuple with a join key *v* exists in $T_{n}$, *n* deletes *v* from *L*. (4) If *L* is not empty, *n* broadcasts *L* to its children.

This process continues down the routing tree until *L* becomes empty. *L* may have a join key *w* due to false positives of the BF. In the step (3), *w* may be deleted even when *n* is with the BF flag = *U*. In the worst case, *w* is deleted when a node with the BF flag = *NU* is reached. In such a node, *w* is not found in any set of the join keys in its subtree synopsis. Thus, it is revealed that *w* was included in *L* due to a false positive.

#### 3.2.9. Range Synopses without Bloom Filters

The update of region synopses in searching for the anchor points increases the amount of communication. If no anchor point is found after checking all the pairs of range descriptors in the two region synopses that are initially constructed in the two regions, the update of region synopses is inevitable. The update may be needed more than once. It is desired to conduct as few updates as possible.

In a region synopsis, the join keys is represented in the range descriptors or in the BF. The join keys in the range descriptors are used to search for the anchor points. For early finding of the anchor points, it is good to include as many join keys as possible in the range descriptors. Considering this, in the root of the routing tree, it may be desirable not to use the BF, even when the region synopsis is not to be completely constructed without a BF. The purpose is to allocate more space to the range descriptors. In this case, the BF flag is set to needed but not used (*NNU*). This exception can be applied to some nodes at the top levels of the routing tree, not just to the root.

Reflecting these changes, Observations 2 and 3 need to be slightly modified: Originally, it suffices to access only the node *n* in question. Now, it may be that the BF flag = *NNU* in *n* and in some of its descendants. Thus, it might be necessary to access not just *n* but additional nodes along each path from *n* up to the first descendant of *n* with BF flag = *U* in each path. The join filtering and the tuple search described earlier are accordingly modified.

## 4. Optimization

In this section, we solve some optimization problems for our protocol. In Section 4.1, we figure out the optimal length of the Bloom filter in a region synopsis. In Section 4.2, we come up with the optimal size of a range of the skyline attribute, where the size of a range is defined as the number of tuples that belong to the range.

### 4.1. The Optimal Length of the Bloom Filter

The capacity of the RAM in a sensor node is usually very small unless the mote is an expensive one. When setting up large-scale WSNs, the expenditure for a large number of motes could be very high. In this paper, we deal with the case where the memory space of each sensor node that can be allocated for storing a range synopsis is very constrained.

**Figure 10:**
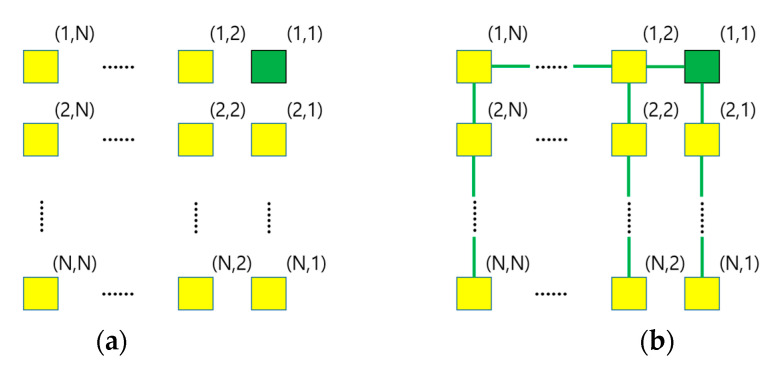
Region of a sensor network and its routing tree. (**a**) A region that consists of *N* × *N* nodes. (**b**) Comb-type routing tree with the root node at (1,1).

We consider a WSN where sensor nodes are deployed in a grid. A region of the WSN consists of $N^{2}$ nodes that are in an *N* × *N* grid as shown in Figure 10a. Figure 10b shows a comb-type routing tree in each region. The node at (1,1) represented by a green rectangle in the upper right corner, is the root. This node has two children: (1,2) and (2,1). The green lines between nodes denote the wireless connection between a parent and a child. There is one horizontal path (1,1)-(1,2)-… -(1,*N*), and *N* vertical paths (1,i)-(2,*i*)-…-(*N*,*i*), *i* = 1,…, *N*. For each vertical path, let us call the node on the horizontal path as *head* and the sequence of remaining nodes as *tail*.

We assume that the BF is not installed in the head node for the reason described in Section 3.2.9. Only the nodes in the tail can have a BF in case of memory overflow. For each vertical path, we figure out the optimal length of the BF in the child of the head. The number of join keys collected in this node is the largest among the nodes in the tail.

In the tail of each vertical path, there are a total of *N*−1 tuples. In the worst case in terms of the space needed to store the range synopsis for these tuples, the ranges to which each of these tuples belongs are different from one another. Let us consider this worst case. In the tail of each vertical path, there are a total of *N*−1 join keys. Suppose $K_{r}$ keys are represented in the range descriptors, and the remaining $K_{b}=$ *N*−1$-K_{r}$ keys are inserted into the BF. The space *b* for storing all the data items of a subtree synopsis that are depicted in Figure 6 and Figure 7, except the BF is as follows:

$$b=s_{c}^{rd}+\left(s_{ID}+s_{c}^{jk}+s_{jk}\right)\cdot K_{r}+s_{w}$$

where $s_{c}^{rd}$, $s_{ID}$, $s_{c}^{jk}$, $s_{jk}$, and $s_{w}$ are the sizes of RDC, RID, JKC, a join key, and the watermark, respectively. All these sizes are in byte. The bits for the BF flag are ignored or a few bits of RDC are assumed to be reserved for the flag.

From the formulas for the probability of a false positive and for the optimal number of hash functions in the Bloom filter theory [38,39] presented in Section 2.3, the optimal length *m* of the BF is obtained with the following formula when the acceptable probability of a false positive is set to *p*:

$$m=-\frac{k_{b}\cdot \ln p}{{\left(\ln2\right)}^{2}}.$$

To evaluate this formula, we need to know the optimal value of $K_{b}$. Since $K_{b}=$ *N*−1$-K_{r}$, we need the optimal value of $K_{r}$. Let *B* be the space allocated for storing a subtree synopsis in each node. The space for the BF would be B− *b* bytes, i.e., $8\cdot \left(B-b\right)$ bits. Since $K_{b}$ join keys are inserted into the BF, the following holds:

$$8\cdot \left(B-\left(s_{c}^{rd}+\left(s_{ID}+s_{c}^{jk}+s_{jk}\right)\cdot K_{r}+s_{w}\right)\right)=-\frac{\left(N-1-K_{r}\right)\cdot \ln p}{{\left(\ln2\right)}^{2}}.$$

Thus, we have the optimal value of $K_{r}$ as follows:

$$K_{r}=\frac{B-s_{c}^{rd}-s_{w}+\left(N-1\right)\cdot \frac{lnp}{8{\left(ln2\right)}^{2}}}{\frac{lnp}{8{\left(ln2\right)}^{2}}+s_{ID}+s_{c}^{jk}+s_{jk}}.$$

Note that the value of *m* computed with the above formula may not be an integer. Thus, the optimal value of *m* is set to the smallest integer that is a multiple of 8 and greater than or equal to the outcome of the formula.

### 4.2. The Optimal Size of a Range

The size of a range *r* is defined as the number of tuples that belong to *r*. In other words, it is the number of join keys that are included in the set of join keys in the range descriptor of *r*. Assuming that the skyline attribute values are uniformly distributed in the tuples sampled in each region, the size of a range determines how many intervals the domain of the skyline attribute is divided into.

In the case that the size of a range is set to 1, the skyline join query is to be processed after the in-network sorting of the tuples on the skyline attribute. That is infeasible, and thus, the size of a range should be greater than 1. In figuring out the optimal size, the trade-offs described below need to be considered.

If an anchor point is not found with the initial region synopses, they need to be updated. To reduce this cost, we need to reduce the number of updates necessary and to reduce the cost of each update. For the former, the number of join keys in the initial region synopses should be increased with a large size of a range. The more join keys in the region synopses, the earlier an anchor point can be found. For the latter, the node where the collection of new range descriptors starts should be as near the root as possible. The command for the synopsis update does not need to be broadcast further down the routing tree when it reaches the first node *n*, where no memory overflow occurred during the initial construction of the region synopsis. The collection of new range descriptors for the update starts at *n* towards the root. The larger the size of a range is, the higher the space utilization in storing the join keys in the region synopses is, and thus, the closer *n* is to the root. In all, the larger the size of a range, the better in reducing the cost of synopsis updates.

As the size of a range increases, on the other hand, the total number of tuples that need to be searched in WSNs and sent to the sink node increases compared with the case of a smaller range size. Some of these tuples are irrelevant to the query result. However, such extraneou*s* tuples should be considered to guarantee the correctness of the query result. The reason why the number of extraneous tuples increases as the size of a range increases is due to the characteristic of our anchor points employed for skyline filtering. We use the point at the upper right corner of the rectangle representing the range pair *p* where one or more join matches are found as the anchor point, as shown in Figure 9. Suppose that a point in the center of the rectangle is also an anchor point. That one would be more efficient in skyline filtering than the one we use. If that anchor point were used, (k−1)/2 more join keys associated with the range of $S_{i}$ in *p* would be filtered, where *k* is the size of a range, *i* = 1, 2. As *k* increases by 1, roughly 0.5 extraneous join keys per skyline attribute need to be considered in searching for and sending tuples. This means that as the size of a range gets larger, the more overhead in communication will incur.

We consider a WSN where each region consists of $N^{2}$ nodes that are in an *N* × *N* grid with a comb-type routing tree as shown in Figure 10. The optimal size of a range is obtained as follows:

#### 4.2.1. The Cost of Region Synopsis Update

Let $C_{su}\left(k\right)$ denote the cost of a region synopsis update in each region when the size of a range is *k*. To compute this cost, we need to know at which node *n* in each tail of the routing tree, memory overflow occurred *first* during the initial construction of the region synopsis. For each vertical path in the routing tree of Figure 10b, let the level of the head be 1, and the levels of the nodes in the tail be 2, 3, …, *N* down the routing tree. The level where *n* is located directly affects $C_{su}\left(k\right)$.

In each region, there are $N^{2}$ nodes, and thus, the number of all the ranges R is $\left\lceil N^{2}/k\right\rceil$ when the size of a range is *k*. Let us consider a subtree *t* consisting of *u* nodes. There are *u* skyline attribute values in *t*. Suppose they belong to a total of *v* different ranges (*v* ≤ *u*). Since the probability that a certain range is not among the ranges for these *u* skyline attribute values is ${\left(1-1/R\right)}^{u}$, *v* is estimated as $\left\lceil R\cdot (1-{\left(1-1/R\right)}^{u})\right\rceil$. There are *v* range descriptors in the subtree synopsis of *t*. Let *B* be the space allocated for storing a subtree synopsis in each node, and *S* be the size of the subtree synopsis of *t*. The condition that no memory overflow occurs in storing the subtree synopsis of *t* is *S* ≤ *B*. Let $u_{max}$ be the maximal *u* that satisfies this condition. Then, for each vertical path of the routing tree, the node where memory overflow occurs first during the initial construction of the region synopsis, is at level $N-u_{max}$. Thus, the level of the node where the collection of new range descriptors starts in a synopsis update is $L\left(k\right)=N-u_{max}+$1 when the size of a range is *k*.

We define the cost of query processing as the number of packets transmitted in WSNs. $C_{su}\left(k\right)$ is the sum of $P_{1}$ and $P_{2}$ below, where *P*(*s*) denotes the number of packets to send *s* bytes.

$$P_{1}=\left(d_{m}+N\cdot \left(L\left(k\right)-1\right)\right)\cdot \;P\left(s_{m}\right)$$

$$P_{2}=\left(d_{m}+N\;\cdot \;L\left(k\right)-1\right)\cdot \;P\left(s_{m}+B\right)$$

If no anchor point is found at $\tilde{m}$, $\tilde{m}$ commands each region to update its region synopsis. $P_{1}$ is the total number of packets sent to deliver this command, where $d_{m}$ is the distance from $\tilde{m}$ to $r_{i}$ in the number of hops, *i* = 1, 2. Every command is contained in a message, and $s_{m}$ is the size of a message. $P_{2}$ is the total number of packets sent during the update and delivery of the new synopsis to $\tilde{m}$. It is assumed that during the update, each node of the routing tree involved sends as much as the maximum bytes possible (i.e., *B* bytes) to its parent. The same from $r_{i}$ to $\tilde{m}$, *i* = 1, 2.

#### 4.2.2. The Cost of Searching and Transmitting of Extraneous Tuples

Let $C_{et}\left(k\right)$ denote the cost of searching and transmitting of extraneous tuples in each region when the size of a range is *k*. Assuming that the number of extraneous join keys $k_{e}$ is (k−1)/2 as described earlier, the total number of extraneous tuples $T_{e}$ in each region with the join rate $J_{r}$ reflected is $2\cdot k_{e}\cdot J_{r}$. Thus, $C_{et}\left(k\right)$ is the sum of $P_{3}$ and $P_{4}$ below:

$$P_{3}=T_{e}\lceil \frac{N-1}{2}\rceil \cdot \;P(s_{m}+s_{jk})$$

$$P_{4}=T_{e}\cdot \;(2\cdot \lceil \frac{N-1}{2}\rceil \;+d_{b})\;P(s_{m}+s_{t}).$$

It is assumed that the node where an extraneous tuple is stored is located in the middle of each region on average. $P_{3}$ is the total number of packets sent to search for the extraneous tuples when the join filter is injected into each region by $\tilde{m}$ to search for all the necessary tuples. $P_{4}$ is the total number of packets sent to transmit the extraneous tuples to the sink node, $d_{b}$ is the distance from $r_{i}$ to the sink node in the number of hops, *i* = 1, 2, and $s_{jk}$ and $s_{t}$ are the size of a join key and that of a tuple, respectively.

#### 4.2.3. The Optimal Size of a Range

Each region sends a range descriptor one by one to $\tilde{m}$ until at least one anchor point is found. The total number of range descriptors transmitted multiplied by the range size gives the total number of join keys transmitted. Let *A*(*k*) be the expected number of join keys that need to be transmitted from each region until an anchor point is found.

Since there are $N^{2}$ join keys in each region, there are $N^{4}$ join key pairs in the Cartesian product between the join keys in the two regions. When the join rate is $J_{r}$, the number of points that correspond to a join match is $N^{2}\cdot J_{r}$. Assuming that these points are uniformly distributed in the 2-dimensional space, the number of join key pairs in the Cartesian product per point is $N^{2}/J_{r}$. *A*(*k*) is estimated as $\sqrt{N^{2}/J_{r}\;}$, which is the length of a side of the square two of whose corners are at the origin and at the anchor point.

The maximum number of join keys that can be stored in a region synopsis is realized when the BF is not installed as described in Section 3.2.9. Let *M*(*k*) be this maximum when the size of a range is *k*. *M*(*k*) is computed as follows, where the notations used are those introduced in Section 4.1:

$$M\left(k\right)=\lfloor \frac{B-s_{c}^{rd}-{\;s}_{w}}{s_{ID}+s_{c}^{jk}+k\;\cdot s_{jk}}\rfloor \;\cdot \;k.$$

In the case that $M\left(k\right)$ is not less than *A*(*k*), the region synopsis in each region needs to be constructed only once. Otherwise, the update of the region synopsis is required, maybe more than once. The number of times $n_{su}\left(k\right)$ is estimated as *round*($A\left(k\right)/M\left(k\right)$) when the size of a range is *k*. Now, the optimal size of a range k* is obtained as follows:

$$k*=\underset{k_{\min}\;\leq \;k\;\leq \;k_{\max}}{\mathrm{minarg}}\left\{\;n_{su}\left(k\right)*C_{su}\left(k\right)+C_{et}\left(k\right)\;\right\}$$

where $k_{min}$ and $k_{max}$ are the minimum and the maximum value of *k* possible, respectively. In theory, $k_{min}\;$= 1 and $k_{max}$ is the number of join keys that can be included in a region synopsis when there is only one range descriptor in it without a BF.

## 5. Simulations

In this section, we report the results of performance evaluation where our protocol SKYJ_W_ is compared with other protocols for skyline join query processing. Referring to [2], the datasets for random, correlated, and anti-correlated distributions are generated. The performance of each protocol is evaluated in terms of the total number of packets sent for query processing in WSNs. The simulation parameters are summarized in Table 1. We considered WSNs where sensor nodes are uniformly deployed in a grid. Each of the two regions for the skyline join operand relations are assumed to be squares consisting of N×N nodes. The number of runs is 100.

In simulations, we set $k_{max}$ described in Section 4.2.3 to a value a bit lower than its theoretical maximum. We empirically noticed that some practical upper bound for $k_{max}$ is necessary to prevent the memory overflow that could occur as $k_{max}$ gets too large. The rule we apply is that the utilization of the space allocated in a node for storing a region synopsis with only a single range descriptor should not exceed 75%. As for the optimal size of a range, only the multiples of 10 among integers between 10 and $k_{max}$ are considered.

Three protocols named EXTERNAL, SJAF, and JF are employed for comparison with our SKYJ_W_. EXTERNAL is a baseline protocol, whereby each node sends its tuple to the sink node where the query is processed. Thus, no in-network skyline or join filtering is conducted. SJAF and JF are the protocols with in-network filtering capabilities. In SJAF, each node sends only the skyline attribute value and the join key of its tuple to $\tilde{m}$, where the skyline and join filtering is conducted in order to obtain the set of join keys of the tuples in the query result. $\tilde{m}$ sends this set to each region, where the tuples whose join keys are in this set are searched and sent to the sink node. In JF, each node sends only the join key of its tuple to $\tilde{m}$, where join filtering is conducted in order to obtain the set of matched join keys. $\tilde{m}$ sends this set to each region, where the tuples whose join keys are in this set are searched and sent to the sink node. The final skyline computation is conducted in the sink node.

### 5.1. Comparison of Protocols for Random, Correlated, and Anti-Correlated Distributions

**Figure 11:**
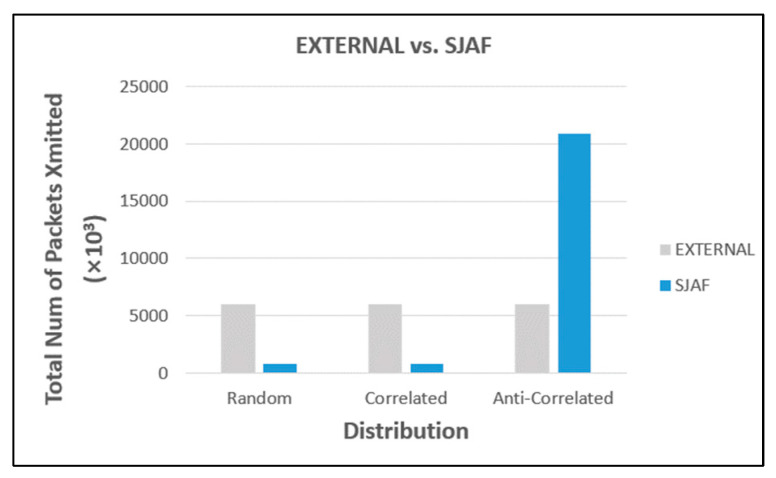
Comparison between EXTERNAL and SJAF.

For each of random, correlated, and anti-correlated distribution, the performance of EXTERNAL and SJAF is compared first. The better one is to be compared with SKYJ_W_ next. Figure 11 shows the result. For random and correlated distributions, SJAF significantly outperforms EXTERNAL (about 87% better for each distribution). The reason is that SJAF conducts in-network filtering, whereas EXTERNAL does not. For anti-correlated distribution, however, EXTERNAL significantly outperforms SJAF (about 71% better). In this distribution, there are so many skyline points. Thus, in SJAF, data communication for skyline filtering results in little effect.

**Figure 12:**
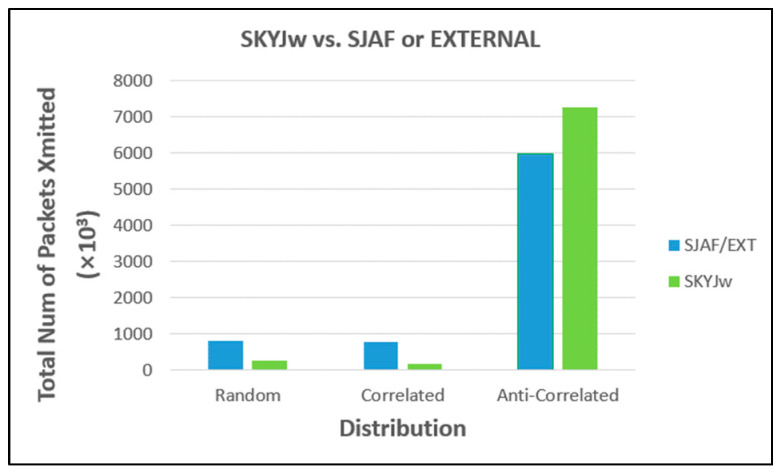
Comparison between SKYJw and SJAF or EXTERNAL.

Figure 12 shows the results of comparing SKYJ_W_ with SJAF for random and correlated distributions, and with EXTERNAL for anti-correlated distribution. SKYJ_W_ significantly outperforms SJAF (about 69% better for random distribution and about 80% for correlated distribution). This shows the effectiveness of in-network filtering of SKYJ_W_. For anti-correlated distribution, however, even SKYJ_W_ is inferior to EXTERNAL. The latter is about 18% better. This inferiority does not imply the ineffectiveness of SKYJ_W_ because there are inherently too many skyline points in anti-correlated distribution, so the skyline filtering effect is naturally not expected to be significant.

As shown so far, the effectiveness of SKYJ_W_ is obvious for the correlated distribution, for which the anchor point could be found early enough. As far as the anti-correlated distribution is concerned, it may not be meaningful to devise an efficient technique of processing a skyline join query when it comes to WSNs. Therefore, in subsequent simulations, the performance of SKYJ_W_ and SJAF is compared only for random distribution.

### 5.2. Comparison between SKYJ_W_ and SJAF for Random Distribution

**Figure 13:**
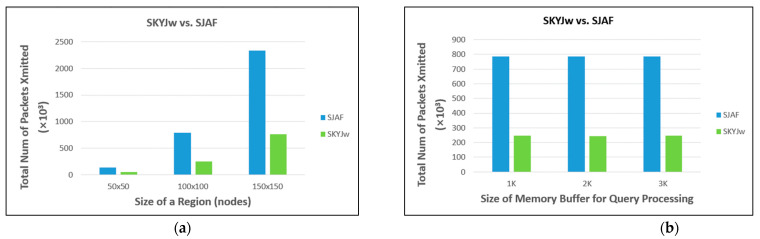
Comparison between SKYJw and SJAF (**a**) as the size of a region varies (**b**) when the size of memory buffer for query processing is constrained.

We vary a few core parameters to evaluate the performance of SKYJ_W_. Figure 13a compares the scalability of SKYJ_W_ and SJAF as the number of nodes in each region increases. The number of nodes in each region is $N^{2}$, and we consider 50, 100, and 150 as the value of *N*. As *N* gets larger, the amount of data transmission increases in both protocols. However, the rate of increase in the number of packets is moderate in SKYJ_W_, but sharp in SJAF. This reveals that SKYJ_W_ is scalable.

Figure 13b examines the effect on the performance of SKYJ_W_ as the memory space allocated for storing a range synopsis in each node varies. While the performance of SJAF is irrelevant to the size of this space, that of SKYJ_W_ might be degraded as the size of this space is reduced. For random distribution, however, the performance of SKYJ_W_ is shown to be little affected. The reason is that the locations of the anchor points are usually not far from the origin of the 2-dimensional space. This means that as far as the random distribution is concerned, efficient in-network query processing is possible using SKYJ_W_ only with a very small amount of memory in each node.

**Figure 14:**
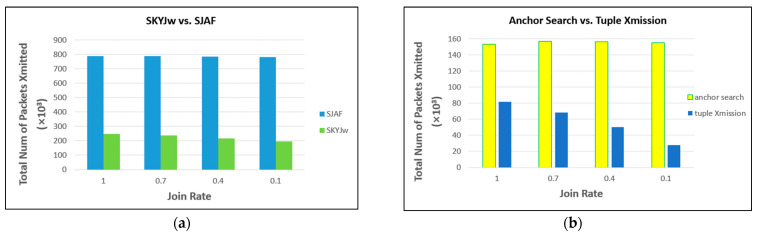
(**a**) Comparison between SKYJw and SJAF as the join rate varies from 1.0 to 0.1. (**b**) The cost for finding the anchor points and that of tuple transmission as the join rate varies as in (**a**).

Figure 14a compares SKYJ_W_ and SJAF as the join rate varies from 1.0 to 0.1. As the join rate gets low, the number of points in the 2-dimensional space decreases, and accordingly, the number of tuples to be transmitted from each region to the sink node decreases. This could result in performance improvement in both protocols. In SJAF, however, the improvement turns out meager. In SJAF, the primary portion of communication cost is for sending the skyline attribute values and the join keys to $\tilde{m}$. This cost is independent of the join rate. In SKYJ_W_, the improvement is significant. In fact, the decrease in the join rate could affect the performance of SKYJ_W_ the other way around as well. It could be degraded because the anchor points get farther from the origin of the 2-dimensional space, and thus, the cost for finding the anchor points would increase. However, the performance degradation turns out to be not prominent, as the join rate gets as low as 0.1. Rather, the factor of aforementioned performance improvement is stronger. Figure 14b shows the cost for finding the anchor points and that of tuple transmission as the join rate varies as in Figure 14a. We can see that the decrease in the latter is more significant.

**Figure 15:**
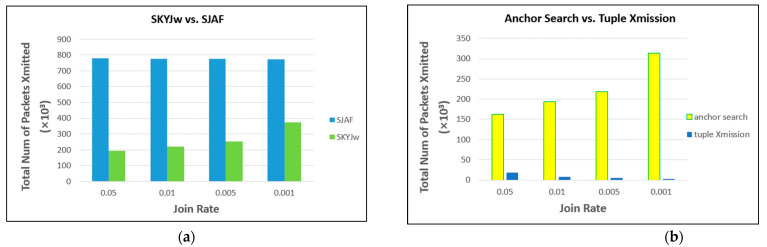
(**a**) Comparison between SKYJw and SJAF as the join rate varies from 0.5 to 0.001. (**b**) The cost for finding the anchor points and that of tuple transmission as the join rate varies as in (**a**).

However, in the case that the join rate gets much lower than 0.1, the increase in the cost for finding the anchor points becomes more significant than the decrease in the cost for transmitting tuples. The performance of SKYJ_W_ gets degraded gradually. Figure 15a compares SKYJ_W_ and SJAF as the join rate varies from 0.05 to 0.001. Figure 15b shows the cost for finding the anchor points and that of tuple transmission as the join rate varies as in Figure 15a. We can see that the increase in the former is much more significant than the decrease in the latter.

**Figure 16:**
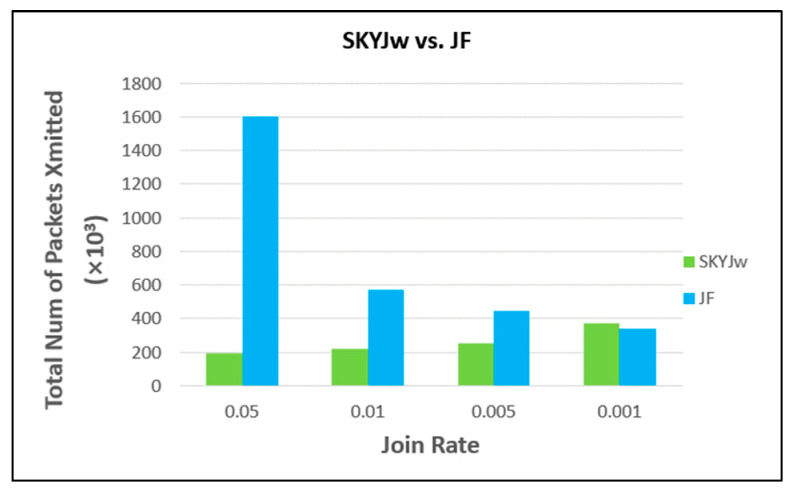
Comparison between SKYJw and JF for very low join rates.

For very low join rates, the protocol JF, which relies on join filtering only without skyline filtering, might be the most efficient. The reason is that the data transmission for skyline filtering is not necessary at all when its portion in the total cost would be larger. However, it is revealed that the join rate should be extremely low in order for JF to outperform SKYJ_W_. Figure 16 compares SKYJ_W_ and JF as the join rate varies from 0.05 to 0.001. We can see that JF outperforms SKYJ_W_ when the join rate is as low as 0.001.

## 6. Related Work

To our best knowledge, no work was reported on skyline join query processing in WSNs. Thus, in this section, we survey various skyline join query processing techniques for the centralized database servers or for distributed databases. These techniques are applied when the skyline attributes are distributed in multiple tables, which may be stored in the same database server or in distributed databases.

The techniques for processing a skyline join query against multiple tables was first investigated in [24]. The methods to integrate representative join algorithms, such as sort-merge and nested loop, into skyline computation were proposed. With the sort-merge join, first the tuples of each table are grouped by the join attribute, and then the tuples that can be judged without join not to be on the skyline are filtered. The remaining tuples are sort-merged for computing the final skyline. With the nested loop join, first the tuples of each table are sorted on a skyline attribute. Then, the joined tuples are obtained in a nested loop in the order of the skyline attribute. Among them, the ones dominated by those already determined to be on the skyline are eliminated.

In [25], the techniques for pruning tuples proposed in [24] were extended for distributed databases. The sort and limit skyline algorithm proposed in [49] was extended to deal with the multiple tables storing the skyline attributes. Another algorithm with iterative pruning was also proposed. Since the tables are stored in different sites of the network, the communication cost is considered in addition to the cost of skyline computation, which is CPU-intensive.

In [26], the techniques for pipelined processing of skyline join queries were investigated. For the type of a join query, the equi-join was considered. For the join algorithms, the sort-merge and the nested loop were considered. In the proposed techniques, the join results are obtained first, and then they are pipelined to the skyline operator.

In [27], a sophisticated technique of pruning the join space that can avoid excessive pairwise tuple-to-tuple dominance checks in processing skyline join queries was proposed. With this pruning capability, two algorithms called skyline-sensitive join and symmetric skyline-sensitive join were proposed to process the skyline join queries over two data sources. In [28], each of the two 2-way skyline join algorithms of [27] was extended to deal with the case in which more than two data sources are joined during skyline processing.

In [30], an algorithm called sort-first-skyline-join was proposed to process a skyline query with a join between two tables. It does not require generating all the join tuples, nor accessing all the tuples of the two join operand tables in computing the skyline. The tuples on the skyline can be obtained progressively while the join is conducted.

Processing of skyline join queries in partitioned distributed databases was investigated in [31,32,33]. In [31], the case where the horizontally partitioned fragments are to be joined for skyline computation was dealt with. In [32,33], the case where the original source table is vertically partitioned with the join attribute being shared by every fragment was dealt with. In [32], it is assumed that each of the vertical fragments is stored in a different server, and that each fragment stores only one skyline attribute. In [33], a generalization of the techniques of [32] was proposed to deal with the case where multiple skyline attributes can be allocated to a fragment. In these works, the join rate was always 1.0 because an original source table is assumed to be vertically partitioned. Additionally, only one anchor point was considered.

In [35], processing of skyline join queries over data streams as well as static data sources were investigated. The proposed SkySuite provides the following two operators: skyline-sensitive join and layered skyline-window-join. The former is for effective processing of skyline join queries in a static environment, and the latter is to deal with a streaming environment by incrementally maintaining skyline join query results over the stream. In [24,34], a skyline join query with aggregate functions were dealt with. In [29], the techniques proposed in [24] for skyline join operations against two tables was extended for an n-way join.

The techniques surveyed so far cannot be directly applied to WSNs. The sensor network database is a distributed database but an extremely partitioned one, where the memory in each node is very constrained. The surveyed techniques based on tuple sorting or grouping cannot be implemented in WSNs. The core operations, such as sorting, are meaningful only when sufficient memory is available. Their complicated data structures and sophisticated bookkeeping for data pruning are not applicable in WSNs. The amount of data transmission required is also not realistic in WSNs. Considering the characteristics of WSNs, the problem dealt with in this paper is quite different from those solved in the previous work.

## 7. Conclusions

In this paper, we investigated the in-network processing of a skyline join query in WSNs. Since the conventional skyline join algorithms for centralized or distributed databases cannot be employed in WSNs, we proposed a protocol that uses the synopses of skyline attribute value ranges. We solved some optimization problems for our protocol. The optimal length of the Bloom filter, which is part of the range synopsis, and the optimal size of a skyline attribute value range can be obtained. A detailed set of simulations showed that our protocol is very effective despite the severe constraints in WSNs.

The range synopsis turns out to be a very compact data structure that makes our protocol work well with the limited memory and energy in each sensor node. For the correlated and random distributions, our protocol significantly outperforms other possible protocols, confirming the effectiveness of in-network skyline as well as join filtering capabilities of our protocol.

As the future work, we plan to extend the simulation model. We considered only reliable environments in the simulations assuming that no packet is lost and no sensor node is disconnected. Our protocol needs to be evaluated further in the presence of these issues. We also plan to figure out the performance characteristics of our protocol further when the cost of finding the anchor points for skyline filtering varies.

## Figures and Tables

**Table 1:** Parameter settings in the simulations.

Parameter	Value
The number of hops between $r_{i}$ and the sink node (*i* = 1, 2)	50
The number of hops between $r_{i}$ and $\tilde{m}$(*i* = 1, 2)	30
Size of a region (*N*×*N* nodes)	*N* = 50, 100, 150
Memory space allocated for storing a range synopsis per node	1K, 2K, 3K bytes
Join rate	1.0, 0.7, 0.4, 0.1, 0.05, 0.01, 0.005, 0.001
Size of a tuple	100 bytes

## Data Availability

Not applicable.
